# Medical comorbidities of patients presenting to an adult neurodevelopmental new case clinic in Singapore

**DOI:** 10.1192/bjo.2021.782

**Published:** 2021-06-18

**Authors:** Ho Teck Tan, James Patrick Moon, Giles Ming Yee Tan

**Affiliations:** 1 Institute of Mental Health, Singapore; 2 Sengkang General Hospital

## Abstract

**Aims:**

To describe the occurrence of medical comorbity in patients with neurodevelopmental disorders presenting to the Adult Neurodevelopmental Service (ANDS) multi-disciplinary new case clinic at the Institute of Mental Health (IMH) in Singapore. We hypothesize that patients with neurodevelopmental disorders have higher rates of medical comorbidity compared to those without a diagnosis of neurodevelopmental disorder.

**Background:**

Medical comorbidities are common in patients with neurodevelopmental disorders. They may have difficulties managing their medical conditions which could in turn affect their well being, quality of life and life expectancy.

**Method:**

A retrospective cohort study was conducted amongst patients who presented to the clinic from January 2015 to December 2016. The electronic case records of the assessments were de-identified and the medical conditions of patients were collected and analysed.

**Result:**

319 patients attended the ANDS new case clinic in the 2-year study period. 87.1% (278/319) were diagnosed with a neurodevelopmental disorder while 12.9% (41/319) did not receive any diagnosis of a neurodevelopmental disorder.

58.3% (162/278) of patients with a neurodevelopmental disorder had at least 1 medical comorbidity while only 31.7% (13/41) of patients with no neurodevelopmental disorder had at least 1 medical condition.

Patients with neurodevelopmental disorders had higher rates of epilepsy (12.2% vs 4.9%), cerebral palsy (3.2% vs 0%) but lower rates of having other neurological conditions (1.4% vs 7.3%) compared to those with no neurodevelopmental disorders.

Patients with neurodevelopmental disorders had higher rates of diabetes (6.1% vs 2.4%), hypertension (6.1% vs 2.4%), hyperlipidaemia (7.1% vs 2.4%) and cardiovascular conditions (2.9% vs 0%) than those without a neurodevelopmental disorder.

In terms of other medical comorbidities, patients with neurodevelopmental disorders had higher rates of thyroid abnormalities (4.7% vs 2.4%), respiratory problems (7.6% vs 2.4%), musculoskeletal conditions (5.8% vs 0%), eye issues (5% vs 2.4%) and hearing problems (2.9% vs 0%) but similar rates of dermatological conditions (10.1% vs 9.8%) and gastrointestinal conditions (4.7% vs 4.9%) compared to those with no neurodevelopmental disorders.

**Conclusion:**

Patients with neurodevelopmental disorders have significantly highly rates of medical comorbidity than those without any neurodevelopmental disorders. This study highlights the need to raise awareness of the common medical comorbidities in patients with neurodevelopmental disorders and to ensure adequate screening and referral for follow-up medical care for them.

